# What will it take? Using an implementation research framework to identify facilitators and barriers in implementing a school-based referral system for sexual health services

**DOI:** 10.1186/s12913-020-05147-z

**Published:** 2020-04-07

**Authors:** Emily Leung, Kathryn J. Wanner, Lindsay Senter, Amanda Brown, Dawn Middleton

**Affiliations:** 1 Cicatelli Associates, Inc. (CAI), 505 Eighth Ave. Suite 1900, New York, NY 10018 USA; 2 Franklin and Marshall College, College Square 931 Harrisburg Ave. 1st Floor, Lancaster, PA 17603 USA

**Keywords:** Consolidated framework for implementation science, School health, Implementation readiness, Implementation tools, Case study methods, Implementation science, School-based referral system

## Abstract

**Background:**

Across the United States, sexually transmitted infections and unintended pregnancy rates are alarmingly high among youth. Schools, due to their proximity and access to youth, can increase student access to sexual health services (SHS) by creating referral systems (RS) to link students to school- and community-based SHS. From 2013 to 2018, the Centers for Disease Control and Prevention’s Division of Adolescent and School Health funded 17 Local Education Agencies (LEA) to partner with priority schools and stakeholders to develop and implement RS to increase student access to SHS. Cicatelli Associates Inc. (CAI) was funded to provide capacity-building to LEA. In 2016–2017, CAI conducted case studies at two LEA, both large and urban sites, but representing different geographical and political contexts, to elucidate factors that influence RS implementation.

**Methods:**

Nineteen LEA and community-based healthcare (CBH) staff were interviewed in the Southeastern (*n* = 9) and Western U.S. (*n* = 10). Key constructs (e.g., leadership engagement, resources, state and district policies) across the five domains of the Consolidated Framework for Implementation Research (CFIR) framework guided the methodology and analysis. Qualitative data was analyzed using the Framework Method and contextual factors and themes that led to RS implementation were identified.

**Results:**

Interviewees strongly believed that school-based RS can decrease STI, HIV and unintended pregnancy and increase students’ educational attainment. We identified the following contextual key factors that facilitate successful implementation and integration of an RS: enforcing state and district policies, strong LEA and CBH collaboration, positive school culture towards adolescent health, knowledgeable and supportive staff, leveraging of existing resources and staffing structures, and influential district and school building-level leadership and champions. Notably, this case study challenged our initial assumptions that RS are easily implemented in states with comprehensive SHS policies. Rather, our conversations revealed how districts and local-level policies can have significant impact and influence to impede or promote those policies.

**Conclusions:**

Through the use of the CFIR framework, the interviews identified important contextual factors and themes associated with LEAs’ implementation barriers and facilitators. The study’s results present key recommendations that other LEA can consider to optimize integration of RS-related evidence-based practices, systems, and policies in their districts.

## Background

Across the United States (U.S.), sexually transmitted infection (STI) and unintended pregnancy rates are alarmingly high for our nation’s youth; young people aged 15–24 acquire half of all new STIs [1]. Among this population from 2013 to 2017, syphilis cases have nearly doubled, gonorrhea cases have increased by 67%, and chlamydia remains the most common STI, with 45% diagnosed cases occurring among 15–24 year old females [2]. Untreated, the sequalae of STIs include pelvic inflammatory disease, infertility, and cervical cancer [3, 4]. Additionally, among adolescents age 15–19, the unintended pregnancy rate is 41 per 1000 females, which is the second highest rate across all age groups [5]. Since almost 40% of high school students reported having engaged in sexual activity, the need for consistent and comprehensive STI and unintended pregnancy prevention services is high [6].

Schools play a critical role in improving access to sexual health services due to their proximity and access to youth. In the U.S., schools have direct contact with more than 15 million students attending grades 9–12 for at least 6 h a day during the key years of their social, physical, and intellectual development [7]. As identified by the U.S. Department of Health, improving access to sexual health services within school settings is “crucial” to eliminating disparities in reproductive health outcomes [8]. In recent years, school-based sexual health services, such as STI screenings, and School Based Health Centers (SBHC) that offer sexual and reproductive health services, have shown promise in increasing positive adolescent sexual health outcomes, including healthy sexual behaviors, contraceptive use, and decrease in unintended pregnancies [9–11]. However, there are myriad barriers and challenges to comprehensive access. While the number of SBHC is increasing, many schools, especially in rural areas, do not have SBHC, and many SBHC have limited or no provision of sexual health services [12]. Only 37% of SBHC are capable of distributing contraceptives onsite; almost 50% are prohibited from doing so by state or local policy, and even among schools that provide sexual health services, utilization of the services may be low [13].

One way for schools to overcome such barriers and to increase student access is by creating comprehensive referral systems to link students to sexual health services—both school- and community-based [14]. Recent research has shown promising findings for improving school-based referral systems; in one study, high school females who received referrals to sexual health services from school staff were more likely to receive birth control, get tested and/or treatment for STIs, and receive an HIV test [15]. Rasberry et al. [16] also found that students who received a referral from school staff were three times more likely to get tested for HIV and STIs, after controlling for factors such as sex, age, race/ethnicity, sexual minority status, and having had sex.

In a direct response to these trends, from 2013 to 2018, the Centers for Disease Control and Prevention’s (CDC) Division of Adolescent and School Health funded the *Promoting Adolescent Health through School-Based HIV/STI Prevention and School-Based Surveillance* project. Through a competitive application process in response to CDC’s notice of funding opportunity, 17 Local Education Agencies (LEA) received funding to partner with priority schools and other stakeholders to develop and implement a referral system and associated protocols, resources, and tools to increase student access to sexual health services. Cicatelli Associates, Inc. (CAI), was funded as the Technical Assistance Center to provide tools and capacity building to the LEAs.

**Fig. 1:**
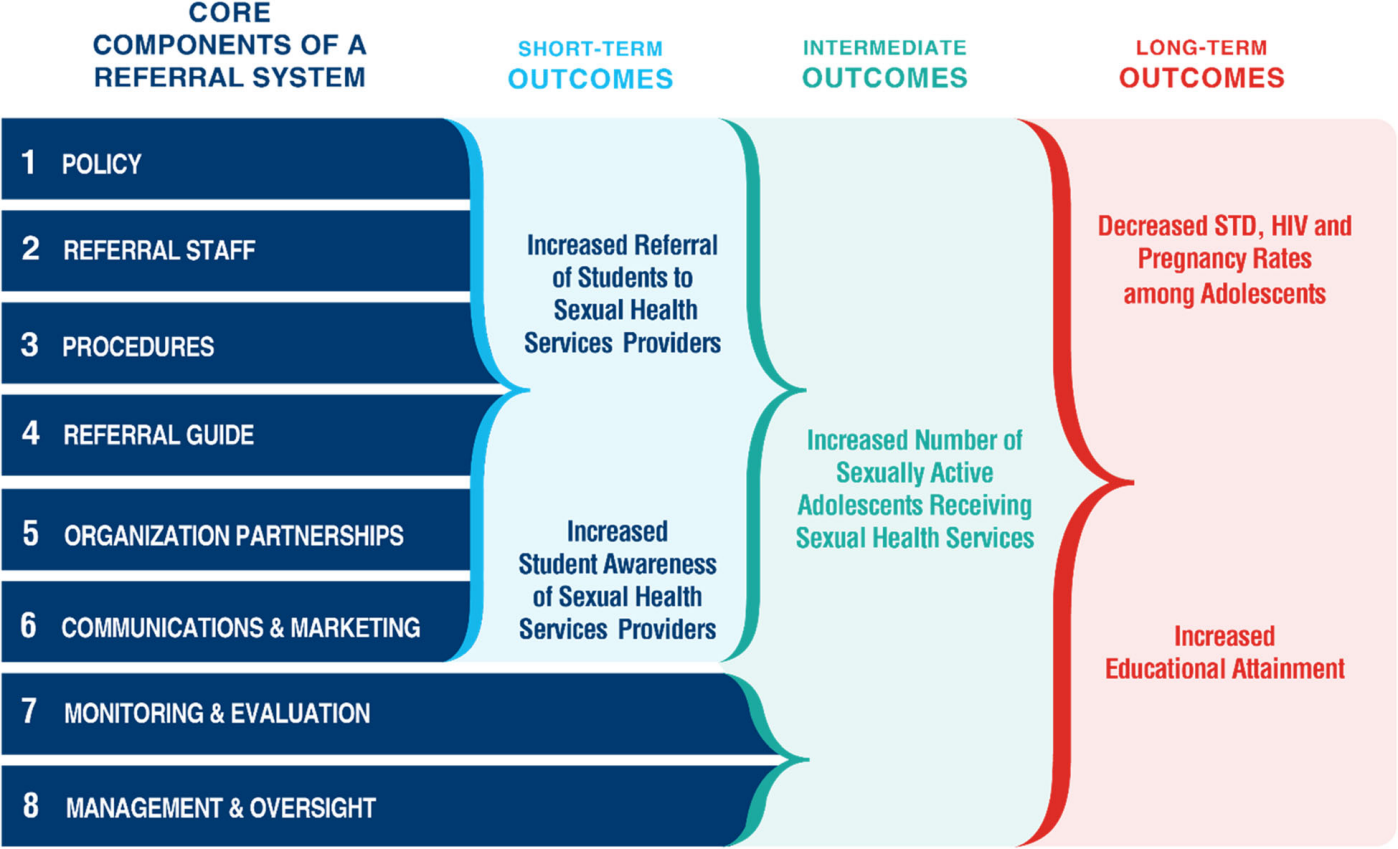
Core Components of a Referral System and Expected Short-term, Intermediate, and Long-term Outcomes

In conjunction with the CDC, CAI developed the Core Components of a Referral System framework that outlines and describes the eight core components necessary for successful referral system implementation (see Fig. 1) [14]. The eight core components include: policy, referral staff, procedures, referral guide, communications and marketing, monitoring and evaluation, management and oversight, and organizational partnerships. *Policies* set forth expectations related to when and under what circumstances students can receive information from school staff about sexual health services, access school-based healthcare, and leave school premises to receive community-based medical services. *Referral staff* recognize adolescents in need of sexual health services and provide referrals. *Procedures* provide a standardized and concise process for school staff to follow to connect students to appropriate sexual health services. A *referral guide* serves as an important tool for staff to select appropriate service providers for students. A *communications and marketing* strategy can help to raise awareness of availability of both school- and community-based adolescent-friendly sexual health services. Establishing a *monitoring and evaluation* plan and system allows for evaluating the extent to which the referral system is achieving its intended objectives. *Management and oversight* ensure that the referral system is being implemented in a standardized manner and is on track to achieve outcomes. Lastly, strategic *organizational partnerships* between schools, school districts, SBHC, health departments, and youth-friendly community-based health providers (CBHP) are needed so that students can access needed services at schools or in the community.

This framework is intended to provide LEA with a standardized approach for implementing a referral system, with the short-term goals of increasing student awareness of school- and community-based sexual health service providers and increasing student referrals to school and community-based sexual health service providers, and the intermediate-term goal of increasing the number of sexually active adolescents receiving key sexual health services. Ultimately, the long-term outcomes of a referral system are decreased STD, HIV, and pregnancy rates among adolescents and increased educational attainment. The framework’s core components do not have to be addressed in any particular order and can be worked on simultaneously or one at a time. Each core component includes a set of recommended activities and tools that LEA can implement, use, and adapt. Throughout the project, CAI provided technical assistance to LEA on implementing the recommended activities and adaptation of tools; however, technical assistance is not required to implement the Core Components of a Referral System.

In the final year of implementation (2017), CAI conducted a case study among two LEA, consisting of in-depth interviews, to investigate contextual factors that could serve as facilitators or barriers to implement a system that connects students to sexual health services in two LEA.

## Methods

### Study design

Data was collected from January to April 2017 using a multiple case study design in two large urban school districts via in-depth qualitative interviews. The authors selected a case study approach, as it allowed for an “in-depth, multi-faceted understanding of a complex issue in its real-life context” [17].

### Application of the consolidated framework for implementation research for interviews

We employed a well-known implementation science framework, the Consolidated Framework for Implementation Research (CFIR) for the case study, as it is essential to consider contextual factors at multiple levels in order for successful integration [18]. The CFIR consists of 39 constructs organized into five domains, that have been used in numerous studies to evaluate and identify key implementation facilitators and barriers [19–21]. The CFIR elucidates important contextual factors necessary for effective implementation of evidence-based practice and policies in real-world settings across five broad areas: intervention characteristics, outer setting, inner setting, characteristics of individuals, and process of implementation (see Table 1).

**Table 1:** Application of CFIR Domains

CFIR Domain	Description and application to current study
1. Intervention Characteristics	Includes characteristics of the referral system and their potential influence on implementation. Examples of constructs selected are: strength of evidence supporting the benefits of implementing an referral system in schools and perceived difficulty implementing referral system (complexity).
2. Outer Setting	Describes the school districts’ external political and social context through which implementation occurs.
3. Inner Setting	Includes the structural, political, and cultural context internally within the school district and school buildings.
4. Characteristics of Individuals	Describes those who are involved in the referral system, such as school-building staff.
5. Process of Implementation	Describes the referral system in its entirety from the planning stage, engaging staff and students, and evaluation.

According to Damschroder et al, researchers may select CFIR constructs that are most relevant to the study setting to investigate, which has been applied successfully in previous studies [22–24]. Prior to developing the interview guides, the research team selected 20 constructs that were most relevant to the study setting and goals (see Additional file 1). CAI developed interview guides with approximately 25 open-ended questions guided and organized by the five CFIR domains and the 20 relevant constructs (see Additional file 2). The Core Components of a Referral System framework was used in the interview guides to ensure standardized definitions and terms describing a referral system (e.g., referral staff) were used. The interview guide was adapted for different groups of interviewees to adjust for relevant differences in roles or tasks.

Two CAI research staff conducted the interviews, each spanning 45–60 min. One interview was conducted via phone while the rest were conducted onsite. CAI digitally recorded and transcribed all interviews and kept files and data in a secure and confidential server during the study period. The interview data was coded using framework method for analysis which was based on all CFIR constructs and domains. The study protocol was reviewed and approved by Western Institutional Review Board and was determined to be exempt.

### Sample

#### Site selection

CAI used convenience sampling to select two large urban school districts located in the Southeastern and Western U.S. for participation. These two LEA reflected geographic diversity, had different operations and policies, and during our tenure working with them as TA specialists, demonstrated varied approaches to implementation that we felt could best inform other LEA throughout the country. Further, the two LEA selected had strong relationships with our technical assistance providers, which ensured they would provide open and honest dialogues about successes and challenges in their work.

#### Recruitment of participants

CAI first contacted each of the LEA’s program coordinators in November 2016 to request their participation in the study. After they agreed to participate, CAI used a snowball sampling technique to select interviewees by asking the program coordinator to identify diverse staff and stakeholders involved with planning, implementing, and evaluating their referral systems, making referrals at their priority schools, or providing referred students with sexual health services. This method of sampling was used since program coordinators were most knowledgeable of who the key stakeholders in implementing the referral system were.

#### Participants

A total of 19 staff members from both districts were interviewed from January to April 2017: 9 staff from the Southeast Site and 10 staff from the Western Site. Interviewees were classified as *district staff* (individuals who worked in the district offices, such as program directors and managers), *school-building referral staff* (individuals who worked directly in schools, such as teachers and school nurses), and *community-based healthcare providers* (CBHP) (individuals who worked at community-based organizations as health educators or as testing coordinators, for example). See Table 2 for types of interviewees interviewed at each site.

**Table 2:** Interviewees by role and site

	Southeast Site (***N*** = 9)	Western Site (***N*** = 10)
**District-level staff** (e.g., program coordinators, evaluators, nursing program manager, school psychologist)	3 (33%)	3 (30%)
**School-building referral staff** (e.g., teachers, nurses, counselors, student coordinators)	3 (33%)	3 (30%)
**Community-Based Healthcare Provider** (e.g., executive director, clinical services director, health educators, HIV/AIDS program coordinators, education and adolescent health manager)	3 (33%)	4 (40%)
**Total**	**9**	**10**

### Analysis

#### Interviews

The “framework method”, integrating both deductive and inductive approaches, guided the qualitative data coding and analysis [25]. All 39 CFIR constructs were used as codes to sort and organize qualitative data as interviewees provided valuable data related to constructs in addition to the pre-selected 20 constructs. Two independent coders coded the data using ATLAS.ti and met to compare and reconcile coding. After coding, common themes by construct and domain were put into a matrix and salient themes were identified for each site as well as across both sites.

## Results

The following findings from the interviews at the two school districts are organized by the CFIR domains and summarized across the two sites and the various staff interviewed. CAI included quotes that demonstrate salient findings, labeled by the interviewees and their role and a randomly-generated identification number. Table 3 provides an overall summary of themes and quotes organized by CFIR domains and constructs.

**Table 3:** Summary of emergent themes from qualitative interviews that influence referral system implementation

CFIR Construct	Themes	Quotations
**I. Intervention Characteristics**		
Evidence Strength and Quality	-Schools are an ideal location to address the epidemic of STIs, HIV, and unintended pregnancy	*“Schools represent… a really important social institution in the lives of young people and means by which we can get young people aware about resources in the community, I think that they’re a key player.”* (Interviewee 07—CBHP)
	-The referral system can help students achieve better academic outcomes	When students can access SHS referrals from school staff, they “*have the ability to focus more on what they are here to do every day, which is to get their education.”* (Interviewee 16—District Staff)
Complexity	-There is a moderate level of difficulty felt when implementing an referral system because of the many layers of bureaucracy	*“It’s such a huge system that is very cumbersome and kind of in a transition mode. So there’s a fair amount of chaos, in my experience, in just dealing with the system”* (Interviewee 3 – CBHP)
**II. Outer Domain**		
Needs and Resources	-There is high need for an referral system because of high STI rates among adolescent populations and adolescents are misinformed about the risks.	“*They’ve all got experience, they’ve all done everything, if you listen to their stories. But they don’t even know basics about female and…. male anatomy.”* Interviewee 14 – School Building Referral Staff)
Cosmopolitanism	-The local health department and CBHP emerged as two main types of crucial partners for these school districts	*“We have a fantastic relationship with the health department…We work hand-in-hand.”* (Interviewee 18 –District Staff)
State and District Policies	-State and district policies can be important facilitators or barriers to implementing an referral system. Furthermore, although supportive policies may be in place, implementation may be hindered by lack of knowledge or awareness of the policies.	*“I think sometimes policy creates, or has the potential to create, barriers because people don’t understand realistically what the policy is saying.”* (Interviewee 16 –District Staff)
**III. Inner Setting**		
Networks and Communications	-Sites drew from existing staffing structures and developed innovative marketing and communication tools (e.g., palm-sized chat cards)	The nursing staff *“are broken into clusters…I think I have more actual discussion of how to handle [SHS referrals] at our cluster level than I do at the district level, just because those are the nurses I see more often, and we have the same student populations.”* (Interviewee 15 – School Building Referral Staff)
Organizational Culture and Access to Knowledge and Information	-Culturally conservative climate impacts the referral system in many ways. Although a school district may be located in a more liberal city, conservative attitudes at the individual level can still affect the referral system	*“It’s such a culturally conservative environment that most people who work with teenagers and young people are really afraid of sexuality, and they’re afraid to really go there. (*Interviewee 3 – CBHP*)*
Leadership Engagement	-Principals have a lot of authority on the day-to-day operation of schools but their acceptance and commitment to implementing a sexual health referral system varies	*“The principals still almost have reign over their school… And the principals can decide the way that things run in their school. And they could be, for instance, homophobic or even sex negative* versus *sex positive.”* (Interviewee 4 – District Staff)
	- District-level leadership (e.g., superintendent) and community-based health service providers were described as supportive, involved, and motivated	*“The leaders here are really on board with [the referral system], and they really encourage us to do a lot more and get the program really out there to do a lot for the students. They’re very supportive”* (Interviewee 8 – School Building Referral Staff)
Available Resources	-A full-time referral staff as crucial in the success of the referral system -SBHC were identified as important resources	*“Knowing that [the referral staff] is there five days a week and knowing the level of conversations that she has with kids …and the time that she spends linking and connecting families to services, I know why kids are doing better in her school.”* (Interviewee 16 – District Staff)
**IV. Characteristics of Individuals**		
Knowledge and Belief	-Staff who were actively involved were most knowledgeable about the logistics of referral-making and the policies. School staff and students have a general knowledge of the staff to refer to for health-related questions.	“…*Our school staff are pretty well-informed about the services that the [SBHC] offers. They may not know how the logistics works off campus…And I don’t think they really know all of the law surrounding [state’s family planning law].”* (Interviewee 19-CBHP)
**V. Process**		
Engaging	-The role of formally appointed implementation leader was essential in supporting implementation, especially in engaging key stakeholders and provision of professional development	*“Well, without somebody advocating for [the SHS referral system] in the school district, I don’t think it would have ever happened. So I give [Program Coordinator] a lot of credit.”* (Interviewee 13—CBHP)
Reflecting and Evaluating	-Tracking referrals was identified as challenging as current data collection systems do not capture passive referrals or efforts that don’t necessarily result in a referral	*“So I think probably…some of the referrals aren’t being tracked the way that they should be. But we don’t really have systems in place that would support that process”* (Interviewee 2-CBHP)

### CFIR domain 1: intervention characteristics

The CFIR domain, *intervention characteristics*, contains constructs that influence the implementation of the referral system (intervention), including evidence strength and quality, and complexity.

#### Evidence strength and quality

This construct investigated the extent to which interviewees felt that a school-based referral system will lead to the expected long-term outcomes—decrease in STI, HIV, and unintended pregnancy and increase in education attainment. Overall, interviewees reported that schools are in fact, an ideal location to implement referral system because students may not be able to access sexual health services at home or in their greater community. As one clinical services staff at a CBHP mentioned, *“some young people don’t have supportive parents, they live in a strict environment…for them it provides an opportunity to be able to get access to sexual health which is so important*” (Interviewee 02-CBHP). In addition, most interviewees felt that a strong referral system can ease students’ stress and anxiety in determining who and where to ask for help, thereby improving students’ ability to focus at school, school attendance, and thus, educational attainment.

#### Complexity

During the interviews, participants overwhelmingly reported that the many layers of bureaucracy and the chaotic nature of the school system make implementation difficult. As one district staff mentioned, *“the challenge we face is our bureaucracy…there’s so many people that have to be involved that sometimes it hinders the process.”* (Interviewee 04-District Staff). Another interviewee cited how “*[The school district] is such a huge system that is very cumbersome and kind of in a transition mode. So there’s a fair amount of chaos”* (Interviewee 03-CBHP).

### CFIR domain 2: outer setting

The second domain, *Outer Setting*, explores external factors that may influence the referral system, such as student needs, state and district policies, and partnerships.

#### Needs and resources

Most of the interviewees believed that connecting students to sexual health services is a high need. As one interviewee states, *“On a scale of one to ten based on how much students need [sexual health services], I’d probably give it an 11”.* (Interviewee 02 -CBHP).

One reason is the high STI rates among the adolescents, specifically where the school districts are located: *“We have a hot spot for chlamydia that we’ve seen, more outbreaks of syphilis than we have seen in the past.”* (Interviewee 02-CBHP). Interviewees also expressed that many students are sexually-active but are misinformed about risks, as they often share incorrect information with each other. Many interviewees expressed specific concerns for disproportionately affected populations, such as students of color and LGBTQ students.

#### Cosmopolitanism

In this context, cosmopolitanism can be defined as the degree to which each LEA is networked with other external organizations [17]. While each site has myriad local and state partnerships, two types of organizations emerged as crucial partners: local health departments and CBHP. Interviewees noted that through the planning and implementation of the referral system, partnerships among these organizations were strengthened. “*It was a tenuous relationship in the past but…this grant has really increased our ability to access the school board and have a seat at the table”* (Interviewee 03-CBHP).

Specifically for local health departments, the interviewees expressed that they played a prominent role in supporting professional development sessions for teachers and staff, offering sexual health services to students through SBHC and assisting LEAs with monitoring and evaluation activities.

CBHP, described as *“youth-friendly, LGBT-inclusive, more up-to-date than typical clinicians on the latest birth control methods and STI testing, HIV testing”* (Interviewee 11-District Staff), also play active and crucial roles in the implementation of the referral system. Interviewees reported their involvement in providing sexual health services to students, delivering training and technical assistance to other CBHP, and in creating resources for LEAs (e.g., referral guides, staff professional development curricula). Interviewees also felt that these partnerships increased CBHP’s awareness of state and local policies regarding minors’ access to health services.

Additionally, interviewees from the Southeastern site mentioned that the relationship between the CBHP and the LEA has strengthened significantly in the last several years. In the past, the CBHP was not allowed on school campuses due to the larger political climate and a restrictive district policy. Several interviewees mentioned that through collaborating to create a referral system, the partnership has been strengthened: “*people in the school system are much more open now and understand that having a relationship with a youth advocacy organization is a strengthening component”.* (Interviewee 03-CBHP).

#### State and district policies

While interviewees identified how some state laws facilitate referral system—the Western site’s state law allows students to access STI services (including HIV testing and treatment) without parental consent, other policies serve as barriers—the Southeastern state’s guidance for abstinence-only education is an obstacle for schools to deliver comprehensive sexual health education, as well as the law that contraceptive counseling cannot be provided on school campus: “*not being able to talk about condoms or any contraceptives in the school is a humongous barrier”* (Interviewee 12-CBHP). Further, the conflict between state- and district-level policies was observed to be a barrier in the Western site; while the state law allows for student access to sexual health services without parental consent, district policy is more restrictive: *“Sexual health services has to be [provided] through a SBHC with a contract with the district. …There are very few [SBHC] in our school district, and each of those has their own contract about what can or can’t be given”* (Interviewee 18-District Staff).

### CFIR domain 3: inner setting

The third domain, *Inner Setting,* refers to the location, structural, social, organization, and cultural characteristics of where the intervention takes place. Constructs that were explored included networks and communications, culture and climate, leadership, and available resources.

#### Networks and communications

Networks and communications refer to the nature and quality of social networks and of formal and informal communications within the school district. Each site had distinct networks and communication systems, drawing from strong and pre-existing staffing structures and innovative marketing and communication approaches, to facilitate referral system implementation. In the Western site, the referral system was supported by nurses across different schools organized into teams called “clusters.” These clusters met monthly and often discussed successes and challenges related to referring students to sexual health services.

In the Southeastern site, the referral system was supported by a palm-sized ‘chat card,’ a communication tool used by staff to refer students to services. Chat cards have information about teen health center locations, types of services offered, and contact information. However, many interviewees from this site noted that the ‘chat cards’ might not be directive enough to link students to sexual health services.

#### Organizational culture and access to knowledge and information

In the Western site, most staff mentioned that the organizational culture is supportive of sexual health prevention services. However, they also mentioned that the county is more conservative, relative to the rest of the state, and that negative attitudes and stigma towards sexual health education and services exist. Interviewees felt that other school staff did not feel capable of promoting the referral system (e.g., linking students to designated referral staff or giving referral guides to students) due to general fears related to parents, legal issues, and general lack of knowledge about the referral system. One interviewee expressed that, *“there is this nervousness that principals and other people have when kids go to confidential appointments when they’re entrusted by their parents to be at school*” (Interviewee 16-District Staff). Similarly, many staff in the Southeastern site highlighted the culturally conservative climate as negatively impacting the referral system.

#### Leadership engagement

Since school principals have a lot of authority on the day-to-day operation of schools, they can influence the degree of referral system implementation. Most interviewees at both sites reported that principals’ acceptance and commitment to implementing a sexual health referral system varies. A few of the interviewees stated that school-building level champions were at times hard to identify due to lack of buy-in from principals. However, district-level leadership (e.g., superintendents) and CBHP were described overall as supportive, involved, and motivated. As one interviewee stated, “*the leaders here are on board with it, and they really encourage us to get the program out there for the students”.* (Interviewee 07-CBHP).

#### Available resources

Many interviewees discussed the importance of having a full-time referral staff as crucial in the success of the referral system. Further, the Southeastern site mentioned that because the district is spread out geographically and that public transportation is limited, their five SBHC are critical resources for students. As one interviewee stated, “*it’s hard for a lot of the students to get to health clinics unfortunately, which is why it’s important that we are in the schools*.” (Interviewee 05-District Staff). Challenges were also mentioned around SBHC, which included space, staffing, inadequate hours, and difficulty for students who attend other schools to access.

### CFIR domain 4: characteristics of individuals

The fourth domain describes the individuals involved with the referral system, such as district staff, school staff, CBHP and leadership. Specifically, these individuals’ knowledge and beliefs regarding sexual health services emerged as a salient construct.

#### Knowledge and belief

Across the two sites, interviewees reported that staff who were the most actively involved in referral system (e.g., referral staff, program coordinator, champions) were the most knowledgeable, especially regarding the logistics of making a referral, policies, and promoting student access to health services. Interviewees stated that knowledge about the referral system is generally higher among all staff and students if the school has a SBHC onsite or if the referral system is heavily marketed through posters, flyers, and/or announcements.

However, interviewees reported that beliefs towards the referral system among school-building staff were mixed. Negative attitudes and the stigma of discussing sexual health came up as major barriers across the two sites. In the Southeastern site, interviewees reported that their colleagues are hesitant to make referrals as they do not want to appear that they are endorsing adolescents having sex. One district staff said: “*…We’re in the Bible Belt and I think some of the mindsets…about the way that children should be behaving…hinder the referral process.”* (Interviewee 04-District Staff). Similarly, in the Western site, one interviewee stated “*Some people are worried about kids leaving campus and they don’t agree with the law and they question the law”* (Interviewee 16-District Staff).

Additionally, many interviewees stated that despite having supporting policies in place at the state- or district-level, lack of knowledge or misunderstanding of the policies are barriers to sexual health service provision. Laws surrounding consent are often difficult for school staff to comprehend because it is outside of typical practice for schools to allow students to do anything without parental consent: “*The school system does not do anything with young people without parental consent and engagement, except for in rare situations”* (Interviewee 03—CBHP). Furthermore, many interviewees mentioned that sometimes, local- and state-policies conflict, which is confusing for school and district staff.

### CFIR domain 5: process of implementation

The last domain looks at the entire process of the referral system through different stages of implementation. Salient constructs in this domain include engaging staff and students in the referral process and reflecting and evaluating.

#### Engaging

Many interviewees spoke of the necessity of the Program Coordinator position to employ different strategies to engage the appropriate individuals to ensure successful implementation and use of the referral system. Examples of these strategies include: recruiting and training referral staff on referral procedures and minor consent and confidentiality laws; meeting with district and school leaders to provide updates on referral system implementation; establishing and maintaining formal partnerships with CBHPs; and distributing communication and marketing materials in schools. As one CBHP manager stated, “*I’ve been really impressed with her ability to sort of navigate all of this…a lot of it just falls on her. She’s really coordinating with everyone and working with the schools and coordinating with the partner agencies.”*

Specifically, professional development proved to be important to engage designated referral staff (e.g., counselors, school nurses), to ensure that they know about Core Components of a Referral System and the health resources available for students, and to increase their knowledge in local STI, HIV, and pregnancy statistics.

Engagement of students mostly occurred through posters, flyers, palm cards, school announcements, banners, and calendars. Social media approaches (e.g., Instagram, Snapchat) are currently being developed as well in these sites.

#### Reflecting and evaluating

Methods to conduct ongoing tracking and evaluating of the referral system varied depending on the school district. In the Southeastern site, the local health department develops tracking tools, collates data, and ensures data quality. In the Western site, the program coordinator developed a paper log for nurses to track data and distribute a monthly survey to collect data. Across the two sites, tracking referrals was identified as very difficult as the current data collection systems does not capture passive referrals (e.g., self-referral when student seeks out sexual health services on their own after seeing a poster) or efforts that do not result in a referral. Further, only the staff who were most involved in referral system implementation regularly receive data reports and take the time to use data to evaluate their work.

## Discussion

Overall, this study demonstrates, taking into account inner and outer contexts, that schools can be ideal locations to address STI, HIV, and unintended pregnancy prevention among adolescents. Our findings also indicate that referral systems linking students to sexual health services are capable of being integrated and implemented across different kinds of settings; the Southeastern site implemented their referral system in a less favorable implementation climate (e.g., more restrictive state policies, fewer resources to leverage, etc.), whereas the Western site had a more supportive political climate. The use of an implementation science approach allowed us to identify, explore, and report on factors across multiple domains that influenced implementation at each site, offering a useful framework for other LEA to consider when beginning or continuing to refine a referral system in their districts, regardless of differing policies and/or environments.

A salient facilitator identified within the *outer setting* is the extent of collaboration between schools and external partners, such as community-based healthcare providers. True collaboration, when there was the highest level of partnership, consisted of multiple factors: a shared vision/outcome, shared ownership of the system by health and education partners, and formalized agreements [26]. The study demonstrates how strong partnerships often took the burden off of school staff to be the only ones to refer students to sexual health services, especially if they lacked confidence or capacity to do so. By collaborating and referring out to other organizations, LEA were able to share responsibility and also ensure provision of culturally-competent services. Therefore, inviting community agencies and organizations with similar visions of improving student health and academic outcomes to partner with local school districts is another promising approach for strengthening school-based referral systems [27].

Other influencing factors with the *outer context* domain included statewide policies, which were shown to heavily impact referral system implementation in both settings. Not only did prohibitive policies emerge as a significant barrier, we also found that having supportive statewide policies alone are not always sufficient. Interviewees described how important it is for implementers to be knowledgeable of their districts’ policies in order to make progress in their referral systems.

The importance of being knowledgeable about policies also emerged during questions covering the *inner setting* and *characteristics of individuals* domains. Lack of knowledge regarding policies led to school staff’s avoidance of implementing aspects of the referral system (e.g., not providing referrals or referral guides to students) for fear of being noncompliant with policies. As we saw in the Western site, even in a supportive sexual health environment, school staff who serve as gatekeepers (i.e., principals, office staff, hall monitors) were unaware of the policies of student access to services; this gap in awareness and knowledge has been corroborated in past studies as barriers to sexual health and nutrition education and policy implementation within schools [28–31]. Equally important to increasing staff’s knowledge of state- and district-level sexual health policies is addressing negative attitudes and stigma. Interviewees described how the presence of negative or unsupportive attitudes regarding the referral system, due to societal stigma surrounding teens accessing sexuality education and health services, was a significant barrier to implementation. Acknowledging and addressing stigma and conservative views around sexual health, especially in the beginning phases of implementation, is critical [32, 33]. One way to mitigate these gaps in knowledge and negative attitudes may be to increase guidance and education regarding state and local policies via professional development and trainings in schools.

Facilitators identified within the *inner setting* domain related to identification and leveraging of existing resources, communication networks, and workflows to ensure effective implementation. For example, the Western site employed nurses as referral staff and leveraged the nurse clusters’ well-established shared vision of improving student wellness, formal communication structure, peer collaboration, and feedback and review among peers. Understanding different workflows and where there is a potential for integration involves assessment of school district’s existing networks, systems, and partnerships to the wider community, which could then promote implementation success.

A key barrier identified within the *inner context* was school district bureaucracy. Previous studies have also identified how bureaucratic factors, such as lack of supported staff time to support implementation and/or lack of funding and prioritizing by school administration, are barriers to implementing new school-based policies and health promotion programs [34, 35]. As demonstrated in the case studies, the role of the program coordinator provided crucial oversight and structure for the program, and without funding for this full-time dedicated coordinator position, the referral systems may not have been successfully implemented. Therefore, given that funding is often challenging to secure and hiring new staff is not always feasible, it is recommended that LEA take advantage of already formalized organizational structures, staffing, and workflows where possible.

### Limitations

This study has several limitations to report. First, since we selected only two LEA for inclusion in these case studies, findings may not be generalizable to all LEA throughout the U.S. Also, the interviewees were selected via snowball sampling methods, and therefore, sampling bias may be an issue as the program coordinators may have nominated interviewees who they know well and with whom they have similar traits and characteristics. Another limitation was that the interview guide was developed using 20 selected CFIR constructs, without assessing for all of the CFIR constructs, which could have limited our findings. However, we found that interviewees still provided data related to constructs in addition to the pre-selected 20. Lastly, the perceptions of students, who were the beneficiaries of the referral system, were not explored. Thus, there are likely additional important experiences and perceptions of the referral system not captured by the current study.

## Conclusions

The study’s use of the CFIR offered a helpful framework to investigate two distinct school-based referral systems, enabling us to identify and describe the complexity of barriers and facilitators involved in establishing a school-based referral system. Three general recommendations emerged from the findings which may have applicability for other LEA interested in implementing a referral system. First, LEA would benefit from appointing key stakeholders who are supportive and will lead referral system implementation; these stakeholders include: school leaders (e.g., principles, superintendents) and champions. School leaders can facilitate a culture shift within a school community and champions are considered imperative to get the project up and running and to integrate intervention practices into current school workflows [36]. Second, LEA should evaluate readiness prior to implementing a referral system and identify contextual factors that will serve as barriers or facilitators to implementation. For example, surveys can be distributed to school staff to assess knowledge, skills, self-efficacy, and attitudes towards providing SHS referrals to students and assessments can be performed to evaluate existing resources that can be leveraged. Third and lastly, based on the results the readiness assessment, LEA should identify strategies to address barriers and create action plans to prioritize goals, create actionable steps towards goals, and delineate roles and responsibilities [37, 38]. For example, a professional development plan for school staff may be warranted to increase knowledge of minor consent laws and policies and decrease negative attitudes and stigma towards sexual health services. Applying these recommendations as well as deliberately assessing presence of facilitators and barriers prior to and during implementation, especially in a complicated school environment, is essential to ensuring adequate preparation and readiness and ultimately improving delivery of vital services to students.

## Supplementary information

**Additional file 1.** Consolidated Framework for Implementation Research (CFIR) Domains and Constructs. The table depicts all 39 constructs organized by the 5 CFIR domains as originally published by Damschroder et al. The CFIR constructs with * were selected for the development of the interview guides for this case study. All 39 constructs were used as codes in the analysis phase.

**Additional file 2.** Interview Guide. The interview guide, organized by CFIR domain and interviewee role, was used to conduct the interviews with three types of interviewees, district-level staff, school-building referral staff, and community-based healthcare providers.

## Data Availability

The qualitative data used and analyzed for the current study are not publicly available as individual privacy may be compromised. Requests from data may be available upon reasonable request from the corresponding author.

## Abbreviations

CAI: Cicatelli Associates Inc.
CDC: Centers for Disease Control and Prevention
CBHP: Community-based healthcare provider
CFIR: Consolidated Framework for Implementation Science
HIV: Human immunodeficiency virus
LEA: Local education agency
SBHC: School-based health center
STI: Sexually transmitted infections
U.S.: United States
