# Supplementary material for: What will it take? Using an implementation research framework to identify facilitators and barriers in implementing a school-based referral system for sexual health services
Source: BMC Health Serv Res. 2020 Apr 7;20:292. doi: 10.1186/s12913-020-05147-z (PMC7140539; doi:10.1186/s12913-020-05147-z)
Supplement: Supplementary file 2 — Additional file 2. Interview Guide. The interview guide, organized by CFIR domain and interviewee role, was used to conduct the interviews with three types of interviewees, district-level staff, school-building referral staff, and community-based healthcare providers. [file 12913_2020_5147_MOESM2_ESM.docx]

**Appendix B. Interview Guide**

1. **Overview of school district’s Referral System (1 question)**

Q1. To begin our conversation, I would like to learn more about you and your role in the district/school/or organization and how you have been involved in implementing an SHS referral system. What is your professional title and what are your specific roles and responsibilities within your organization?

1. **Referral System Characteristics (2-3 questions)**

| **CFIR Construct** | **District-level Staff** | **School-building Referral Staff** | **Community-Based Healthcare Provider** |
| --- | --- | --- | --- |
|  | Now, we are going to discuss some of your overall impressions implementing the SHS referral system. I also want to hear about your thoughts on the seven core components of the referral system, and how you have implemented the core components, including the referral tools and resources (e.g., referral guide, procedures for referral making) at each school. | Now, we are going to discuss some of your more general impressions making referrals. I also want to hear about your thoughts regarding the SHS referral tools (e.g., referral guide, procedures for referral making). | Now, we are going to discuss some of your more general impressions of partnering with [*insert school district name*] and being part of the referral system to provide SHS to students. |
| Evidence Strength & Quality | Q1. To what extent do you believe that a school-based referral and linkage system can support students in preventing or treating STD/HIV or pregnancy? As well as contribute to achievement of their academic outcomes? | Q1. To what extent do you believe that a school-based referral and linkage system can support students in preventing or treating STD/HIV or pregnancy? As well as contribute to achievement of their academic outcomes? | Q1. To what extent do you believe that a school-based referral and linkage system can support students in preventing or treating STD/HIV or pregnancy? As well as contribute to achievement of their academic outcomes? |
| Complexity | Q2. Next, I would like to review some of the core components within your current SHS referral system and the difficulty in implementing these core components. On a scale of 1-10, with 10 being the most difficult, from your perspective, how difficult is it to implement a district-wide referral system. Why?   - Probe using the 8 core components; how difficult was it to…   - Develop a specific district Policy about making SHS referrals?   - Identify designated Referral Staff?   - Develop district-specific Referral Procedures?   - Develop a Referral Guide?   - Ensure broad knowledge of Referral systems among staff and students (Communications and Marketing)?   - Track and report information about number of referrals made (Monitoring and Evaluation)?   - Provide coordination and oversight for all planning, implementing, and evaluation activities (Management and Oversight)?   - Establish partnerships with community-based health care providers and health department to promote student access to SHS (Organizational Partnerships)? | Q2. Next, I would like to understand your experience around the referral system. On a scale of 1-10, with 10 being the most difficult, from your perspective, how difficult is it for referral staff to make SHS referrals for students. Why?   - Probe using the 8 core components; how difficult was it to…   - Develop a specific district **Policy** about making SHS referrals (if applicable)?   - Identify &n training **Referral Staff**?   - Develop district-specific Referral Procedures (if applicable)?   - Using the Referral Guide (if applicable)?   - Ensure broad knowledge of Referral systems among staff and students (Communications and Marketing)? (if applicable)   - **Track and report information** about number of referrals made (Monitoring and Evaluation)?   - Provide coordination and oversight for all planning, implementing, and evaluation activities (Management and Oversight) (if applicable)?   - Establish partnerships with community-based health care providers and health department to promote student access to SHS (Organizational Partnerships)? | Q2. Next, I would like to understand your experience around the referral system review. On a scale of 1-10, with 10 being the most difficult, from your perspective, how difficult is it to implement a district-wide referral system. Why?   - Probe using the 8 core components; how difficult was it to…   - Develop a specific district Policy about making SHS referrals (if applicable)?   - Develop district-specific Referral Procedures (if applicable)?   - Develop a **Referral Guide** (if applicable)?   - Ensure broad knowledge of Referral systems among staff and students (Communications and Marketing)? (if applicable)   - Track and report information about number of referrals made (Monitoring and Evaluation)? (if applicable)   - Provide coordination and oversight for all planning, implementing, and evaluation activities (Management and Oversight)? (if applicable)   - Establish **partnerships** with [insert LEA] to promote student access to SHS (Organizational Partnerships)? |
| Adaptability | Q3. What are your thoughts on the extent to which district-wide referral system implementation tools can be easily adapted and applied for each of your priority schools? Why? *or* Why not? | Q3. What are your thoughts on the extent to which district-wide referral system implementation tools can be easily adapted and applied for each of the schools you make referrals at? Why? *or* Why not? | N/A |

**IV. Outer Setting (3 questions)**

| **CFIR Construct** | **District-level Staff** | **School-building Referral Staff** | **Community-based Healthcare Provider** |
| --- | --- | --- | --- |
|  | Next, we are going to explore factors within the larger and external environment (e.g., partnerships, state and district policies, student need) that can possibly influence (both positively and negatively) the implementation of an SHS referral system. Specifically, I would like to hear your thoughts on the social and political contexts in which you are doing your work in developing your SHS policies, procedures, and referral guide. | Next, we are going to explore factors within the larger and external environment (e.g., partnerships, state and district policies, student need) that can possibly influence (both positively and negatively) your ability to make SHS referrals. Specifically, I would like to hear your thoughts on the social and political contexts in which you are doing your work in making SHS referrals. | Next, we are going to explore factors within the larger and external environment (e.g., partnerships, state and district policies, student need) that can possibly influence (both positively and negatively) the implementation of an SHS referral system. Specifically, I would like to hear your thoughts on the social and political contexts in which you are partnering with [*insert school district*] and providing SHS. |
| Cosmopolitanism | Q1. How have your partnerships with the following entities been important in your work in implementing the SHS referral system?   - State Education Agency - Health Department - Priority Schools - Community-based providers of SHS - School-based providers of SHS | Q1. How have your partnerships with the following entities been important in your work in making SHS referrals?   - Local Education Agency (i.e., District) - Health Department - Community-based providers of SHS - School-based providers of SHS | Q1. How have your partnerships with the following entities been important in your work in providing SHS to adolescents?   - Health Department (if applicable) - Local Education Agency (i.e., District) - Local Schools |
| External Policy & Incentives | Q2. What are your impressions of how state and district-level policies, regulations, or mandates support or create barriers to implementation of a SHS referral system?   - Probe with state specific policies (e.g., state policy prohibiting school district staff from making referrals for contraception, state law allowing minors to consent to STI & HIV testing & treatment) | Q2. What are your impressions of how state and district-level policies, regulations, or mandates support or create barriers to implementation of a SHS referral system?   - Probe with state specific policies (e.g., state policy prohibiting school district staff from making referrals for contraception, state law allowing minors to consent to STI & HIV testing & treatment) | Q2. What are your impressions of how state and district-level policies, regulations, or mandates support or create barriers to implementation of a SHS referral system?   - Probe with state specific policies (e.g., state policy prohibiting school district staff from making referrals for contraception, state law allowing minors to consent to STI & HIV testing & treatment) |
| Patient Needs & Resources | Q3. To what extend do you think that the students in your district/school need SHS?   - Probe for STD, HIV, and pregnancy prevention needs | Q3. To what extend do you think that the students in your district/school need SHS?   - Probe for STD, HIV, and pregnancy prevention needs | Q3. To what extend do you think that the students in [insert LEA] need SHS?   - Probe for STD, HIV, and pregnancy prevention needs |

**V. Inner Setting (4-5 questions)**

| **CFIR Construct** | **District-level Staff** | **School-building Referral Staff** | **Community-based Healthcare Provider** |
| --- | --- | --- | --- |
|  | Now, we are going to focus on factors at the district- that can influence the implementation of an SHS referral system, such as the inner working relationships between staff and your district’s leadership engagement. | Now, we are going to focus on factors at the school-level that can influence the referral making process, such as the inner working relationships between staff and your schools’ leadership engagement. | Now, we are going to focus on factors at the organizational-level that can influence your partnership with [*insert school district*]. These characteristics include the inner working relationships between staff, your organization’s leadership engagement and your organization’s data collection methods. |
| Culture | Q1. How does providing students with SHS fit or not fit with the mission of the [*district name*]? | Q1. How does providing students with SHS fit or not fit with the mission of your school? | Q1. How does developing partnerships with schools fit or not fit-in with your agencies strategic goals or mission? |
| Goals & Feedback | Q2. What data or information has been shared with you about successes of the SHS referral system? | Q2. What data or information has been shared with you about successes of the SHS referral system? | Q2. What data or information has been shared with you about successes of the SHS referral system? |
| Readiness for Implementation | Q3. To what extent has the school-based SHS referral system been integrated into the work of the district’s School Wellness Committee (if you have one)? | Q3. To what extent has the school-based SHS referral system been integrated into the work of the School’s Wellness Committee (if you have one)? | Q3. To what extent is providing adolescents with SHS a priority for your agency?   - - Probe: Do you have in place the following: urine screening for STDs, rapid testing for HIV, all contraceptive methods in stock (including LARC methods), confidentiality provisions for youth, provide services regardless of ability to pay, walk-in appointments |
| Leadership Engagement | Q4. How would you describe the involvement of key leaders (district director of health services, director of counseling, school board) in the development, implementation, and improvement of the SHS referral system at the district and school building level? | Q4. How would you describe the involvement of key leaders (e.g., district director of health services, principal) in the development, implementation, and improvement of the SHS referral system at the district and school building level? | Q4. How would you describe the involvement of key leaders (e.g., CEO/Executive Director, organization’s board of directors, other senior managers) in the development, implementation, and improvement of the SHS referral system at the district/schools/your organization? |
| Access to Knowledge and Information | N/A | Q5. To what extent did procedures, guidance and training provided to you in your role as a referral staff prepare you for making referrals? Describe professional development (PD) provided and describe what has been most helpful in the PD received? Probe: What kind of PD has been provided to designated referral staff, like yourself, to increase their capacity to make and track referrals? | N/A |

**VI. Implementation Process (9 questions)**

| **CFIR Construct** | **District-level Staff** | **School-building Referral Staff** | **Community-based Healthcare Provider** |
| --- | --- | --- | --- |
|  | Now, let’s discuss some specific things about how your district implemented the referral system. | Now, let’s discuss some of your experiences with the referral-making process. | Now, let’s discuss the process of how your organization’s partnership with [*insert LEA*] developed and is maintained. |
| Planning | Q1. How helpful have the referral procedures and referral guide been to referral staff in making referrals? How about referral tracking guidance and logs? | Q1. How helpful have the referral procedures and referral guide been to referral staff in making referrals? How about referral tracking guidance and logs? | Q1. Can you describe a few concrete steps you and/or your predecessors took to develop a partnership with [*insert school district*]? |
| Engaging | Q2. To what extent do you think district staff, priority school staff, and community healthcare provider partners are aware of the SHS referral system? | Q2. To what extent do you think district staff, priority school staff, and community healthcare provider partners are aware of the SHS referral system? | Q2. To what extent are key agency leaders/health center staff aware of your partnership with [insert LEA]/schools to implement coordinated referral and linkage system? |
| Engaging | Q3. To what extent do you think students within priority schools are aware of the SHS referral system? | Q3. To what extent do you think students within schools are aware of the SHS referral system? | Q3. To what extent do you think students within schools are aware of the SHS referral system? |
| Engaging | Q4. What strategies do you use to communicate/market the referral system to ensure awareness among district staff, school faculty, and students? | Q4. What strategies do you use to communicate/market the referral system to ensure awareness among district staff, school faculty, and students? | Q4. What strategies do you use to communicate/market the availability of your services to adolescents in local high schools or generally? |
| Engaging | Q5. What kind of professional development has been provided to referral staff in order to increase their capacity to make and track referrals? | See Q. 5 from Section V. | Q5. How has your health center partnered with the school district and/or the priority schools to provide professional development to referral staff in order to increase their capacity to make and track referrals? |
| Formally appointed internal implementation leaders | Q6. Is there someone formally appointed at the school district/school/health center level to oversee and direct all activities associated with planning, implementation, monitoring and improvement of the SHS referral system? Who and please describe their title and duties? | Q6. Is there someone formally appointed at the school district/school/health center level to oversee and direct all activities associated with planning, implementation, monitoring and improvement of the SHS referral system? Who and please describe their title and duties? | Q6. Is there a designated key liaison at the school district and/or schools to promote the SHS referral system? Please describe who and what they do. |
| Champions | Q7. In addition to those formally appointed, are there any people/influencers at the **school district/school/health center** level who are championing implementation of the SHS referral system? Can you describe who those people are and the actions they take? | Q7. In addition to those formally appointed, are there any people/influencers at the **school district/school/health center** level who are championing implementation of the SHS referral system? Can you describe who those people are and the actions they take? | Q7. In addition to those formally appointed, are there any people/influencers at the **school district/school/health center** level who are championing implementation of the SHS referral system? Can you describe who those people are and the actions they take? |
| External Change Agents | Q8. And what about stakeholders or influencers **outside** (e.g., members of the SHAC) of the school district/school/health center that may be championing or supporting implementation of the school-based SHS referral system? Can you describe who they are? | Q8. And what about stakeholders or influencers **outside** of the school district/school/health center that may be championing or supporting implementation of the school-based SHS referral system? Can you describe who they are? | Q8. And what about stakeholders or influencers **outside** of the school district/school/health center that may be championing or supporting implementation of the school-based SHS referral system? Can you describe who they are? |
| Reflecting & Evaluating | Q9. Describe systems that are in place to gather data to see how many referrals have been made and/or completed? What is working well/what is challenging? | Q9. Describe systems that are in place to gather data to see how many referrals have been made and/or completed? What is working well/what is challenging? | Q9. Describe systems that are in place to gather data to see how many referrals have been made and/or completed? What is working well/what is challenging? |

**VII. Characteristics of Individuals (3 questions)**

| **CFIR Construct** | **District-level Staff** | **School-building Referral Staff** | **Community-based Healthcare Provider** |
| --- | --- | --- | --- |
|  | Now, I would like to discuss your perceptions of the individuals involved with the SHS referral system. | Now, I would like to discuss your perceptions of school referral-makers. | Now, I would like to discuss your perceptions of the individuals involved within your organization and how they can influence the provision of SHS to students. |
| Knowledge & Belief about the intervention | Q1. Generally speaking, how would you describe [*insert school district*] staff’s attitudes and beliefs around the SHS referral system/processes? | Q1. Generally speaking, how would you describe designated referral staff’s attitudes and beliefs around making SHS referrals? | Q1. Generally speaking, how would you describe your staff’s attitudes and beliefs around the efficacy of partnering with school district and priority schools to develop and implement an SHS referral system? |
| Self-efficacy | Q2. How confident do you think most school-building referral staff feel about their ability to integrate this work into their regular and current jobs? | Q2. How confident do you think most school referral staff feel about their ability to integrate this work into their regular and current jobs? | Q2. How confident do you feel in your ability to address the unique SHS needs of adolescents? |
| Other Personal Attributes | Q3. How motivated would you say [insert school district] is to implementing and sustaining an SHS referral system for students? | Q3. How motivated would you say designated referral staff are to integrating SHS referral-making into their current job tasks? | Q3. How motivated do you believe your agency or clinics are to partnering with schools to link students to SHS? |

**VIII. Conclusion (4 questions)**

| **District-level Staff** | **School-building Referral Staff** | **Community-based Healthcare Provider** |
| --- | --- | --- |
| Thank you so much for your helpful insights so far. We have just a few more concluding questions. | | |
| Q1. What are the biggest lessons you’ve learned thus far from designing and implementing a school-based SHS referral system? | Q1. What are the biggest lessons you’ve learned thus far from making SHS referrals? | Q1. What are the biggest lessons you’ve learned thus far from partnering with [*insert school district*] to increase student access to SHS? |
| Q2. What would you describe as your biggest achievements related to designing and implementing an SHS referral system? What would you describe as some of the greatest challenges? | Q2. What would you describe as your biggest achievements related to making SHS referrals? What would you describe as some of the greatest challenges? | Q2. What would you describe as your biggest achievements as an organization related to providing SHS to students? What would you describe as some of the greatest challenges? |
| Q3. On a scale of 1-10, with 10 being the most difficult, from your perspective, how difficult do you think it will be to sustain this work implementing an SHS referral system at your district in the absence of funding?   - Probe using the 8 core components; how difficult will it be to sustain…   - Policy   - Referral Staff   - Referral Procedures   - Referral Guide   - Ensuring broad knowledge of Referral systems among staff and students (Communications and Marketing)   - Tracking and reporting information about number of referrals made (Monitoring and Evaluation)   - Providing coordination and oversight for all planning, implementing, and evaluation activities (Management and Oversight)   - Establishing partnerships with community-based health care providers and health department to support achievement of goals (Organizational Partnerships) | Q3. On a scale of 1-10, with 10 being the most difficult, from your perspective, how difficult do you think it will be to sustain this work implementing an SHS referral system at your district in the absence of funding?   - Probe using the 8 core components; how difficult will it be to sustain…   - Policy   - Referral Staff   - Referral Procedures   - Referral Guide   - Ensuring broad knowledge of Referral systems among staff and students (Communications and Marketing)   - Tracking and reporting information about number of referrals made (Monitoring and Evaluation)   - Providing coordination and oversight for all planning, implementing, and evaluation activities (Management and Oversight)   - Establishing partnerships with community-based health care providers and health department to support achievement of goals (Organizational Partnerships) | Q3. On a scale of 1-10, with 10 being the most difficult, from your perspective, how difficult do you think it will be to sustain this work implementing an SHS referral system at your district in the absence of funding?   - Probe using the 7 core components; how difficult will it be to sustain…   - Policy   - Referral Staff   - Referral Procedures   - Referral Guide   - Ensuring broad knowledge of Referral systems among staff and students (Communications and Marketing)   - Tracking and reporting information about number of referrals made (Monitoring and Evaluation)   - Providing coordination and oversight for all planning, implementing, and evaluation activities (Management and Oversight)   - Establishing partnerships with school districts and priority schools to support achievement of goals (Organizational Partnerships) |
| Q4. Did we miss anything? Do you have anything else you would like to share with me that you did not get to say earlier? | Q4. Did we miss anything? Do you have anything else you would like to share with me that you did not get to say earlier? | Q4. Did we miss anything? Do you have anything else you would like to share with me that you did not get to say earlier? |
